# Using artificial intelligence and longitudinal location data to differentiate persons who develop posttraumatic stress disorder following childhood trauma

**DOI:** 10.1038/s41598-021-89768-2

**Published:** 2021-05-13

**Authors:** Damien Lekkas, Nicholas C. Jacobson

**Affiliations:** 1 Center for Technology and Behavioral Health, Geisel School of Medicine, Dartmouth College, 46 Centerra Parkway, Suite 300, Lebanon, NH 03766 USA; 2 Department of Biomedical Data Science, Geisel School of Medicine, Dartmouth College, Lebanon, NH 03766 USA; 3 Department of Psychiatry, Geisel School of Medicine, Dartmouth College, Lebanon, NH 03766 USA; 4 Quantitative Biomedical Sciences Program, Dartmouth College, Lebanon, NH 03766 USA

**Keywords:** Psychology, Human behaviour, Predictive markers, Post-traumatic stress disorder

## Abstract

Post-traumatic stress disorder (PTSD) is characterized by complex, heterogeneous symptomology, thus detection outside traditional clinical contexts is difficult. Fortunately, advances in mobile technology, passive sensing, and analytics offer promising avenues for research and development. The present study examined the ability to utilize Global Positioning System (GPS) data, derived passively from a smartphone across seven days, to detect PTSD diagnostic status among a cohort (*N* = 185) of high-risk, previously traumatized women. Using daily time spent away and maximum distance traveled from home as a basis for model feature engineering, the results suggested that diagnostic group status can be predicted out-of-fold with high performance (AUC = 0.816, balanced sensitivity = 0.743, balanced specificity = 0.8, balanced accuracy = 0.771). Results further implicate the potential utility of GPS information as a digital biomarker of the PTSD behavioral repertoire. Future PTSD research will benefit from application of GPS data within larger, more diverse populations.

## Introduction

Post-traumatic stress disorder (PTSD) is a psychiatric disorder characterized by a constellation of chronic mental, emotional, and behavioral states that occur in response to experiencing or witnessing a traumatic event^1^. Persons with PTSD often experience intrusive and distressing thoughts which may be accompanied by recurring nightmares, flashbacks, and strong negative reactions to sensory stimuli such as sounds and touch^1^. PTSD is highly prevalent within the United States, as it is estimated to occur within 3.5% of the population in any given year^2^. Moreover, additional research based on the U.S. population has also calculated that around 7–8 people out of every 100 will have PTSD at some point in their life, contributing to an annual prevalence rate of around eight million^3,4^. Globally, these rates tend to be lower on average, yet country-specific prevalence rates are heterogeneous (lifetime: 0.3–8.8%, 1-year: 1–8.4%)^5^, in part a function of cultural attitudes, country socio-economic status, mental healthcare access, and environmental exposures. In addition to the high point prevalence, PTSD tends to cause considerable disruption to one’s life, with the majority (approximately 70%) of persons with PTSD experiencing moderate to severe impairment^2^.

Ubiquity and severity notwithstanding, access to mental health care is not equitable^6^, a well-appreciated fact that is heightened in socioeconomically disadvantaged ethnic minority populations^7–9^. In settings where resources are made available, underutilization remains a challenge toward achieving equity^6^. PTSD is documented to have higher incidence in socially disadvantaged groups, thus this combination of limited access and underutilization leaves these groups especially vulnerable. Assuming an ability to surmount mental health treatment barriers, PTSD also suffers from underdiagnosis and misdiagnosis^10,11^—a testament not only to its complex etiological milieu, but its high tendency to manifest with other mental comorbidities^12,13^. Regardless of a metric’s validity, the subjectivity of stressor reporting adds challenge for accurate clinical diagnosis^14^. Given the high point prevalence, severe course, diagnostic obstacles, and overall heterogeneous symptom profile, clinicians and persons experiencing PTSD alike would benefit from novel methods capable of readily detecting PTSD symptomology within short-term, non-invasive, objective, and naturalistic settings.

Emergent passive sensing technologies (i.e., the utilization of passively collected intensive longitudinal data from digital devices to develop predictive models of physiology, thoughts, feelings, and behaviors) offer a promising avenue for development into objective measures of PTSD detection that capitalize on clinically relevant behavioral markers. Within the past few years, the number of studies that have leveraged passive sensing within the predictive mental health space, whether derived from smartphone, wearable device, personal computer, or otherwise, are numerous. These works have shown an ability to utilize data collected from such devices and engineer “digital phenotypes” capable of tracking risk and classifying disorder. Remarkably, reliance solely on such passive information has led to high performance across several complex constructs, including anxiety^15–18^, depression^19–24^, schizophrenia^25–27^, bipolar disorder^28–30^, stress^31,32^, addiction^33,34^, and suicidal ideation^35,36^.

Although a myriad of studies has predicted psychiatric pathology utilizing passive sensing, there has been limited attention directed towards the prediction of PTSD diagnostic outcome. Specifically, most PTSD predictive studies have utilized high-burden data collection procedures rather than passively assessed information^37,38^. Uniquely, one study explored passive sensing utility within a veteran population with PTSD to predict risky behavior from accelerometer gesture detection^39^; however, its utility as a predictor of PTSD diagnosis itself was not explored. Separate from any notions of prediction, the digital phenotyping literature has focused on heart rate variability (HRV), and an abundance of evidence exists for its relationship with PTSD within laboratory settings^40–45^. As far as the authors are aware, only one study, conducted among civilian traffic accident survivors^46^, has leveraged this passive sensing information within a predictive paradigm.

To deploy a biomarker (passive or otherwise) for any prediction-based problem, however, it is important that the “surrogate” or representation of the phenomenon under study is supported by existing clinical definition and theory. Central to the diagnosis of PTSD is what is referred to as “avoidance” symptoms^1^. This factor or cluster consists of behaviors in which the individual actively attempts to avoid both internal and external reminders of their traumatic experience. This includes memories, thoughts, and feelings as well as objects, locations, persons, and interactions. Avoidance can therefore be understood both in terms of cognitive and emotional processing as well as in terms of physical location. The integral nature of avoidance in what is largely a highly heterogeneous disorder has been supported in the literature across disparate traumatized populations including war veterans^47^, vehicle accident survivors^48^, and female assault survivors^49^. Its ubiquity and pivotal role in PTSD symptom maintenance has made it a key target in cognitive behavioral therapy^50^. Chief among PTSD clinical interventions is prolonged exposure (PE) therapy^51^ which operates to disrupt avoidance behaviors through direct and repeated confrontation (both imaginal and in vivo) with feared objects, situations, or locations that represent the traumatic experience. PE therapy is highly effective. A twenty-year meta-analysis across 13 studies (*N* = 658) calculated that PE therapy patients, on average, had a more favorable primary clinical outcome measure post-treatment compared with 86% of non-treatment controls^52^. The performance of this therapy further speaks to the significance of avoidance as a clinically significant behavior.

The physical notions of avoidance behavior, as encompassed within the clinical definition of PTSD, may be described in terms of location and where an individual spends time throughout their daily lives. It is not unreasonable to surmise that daily movement patterns, especially in more severe cases, may define physical avoidance symptomology (situations, places, and/or people) and serve as a potential behavioral biomarker of PTSD. Not only is there clinical and theoretical precedence to do so but measuring daily movement and location information as a surrogate of physical avoidance can be accomplished objectively, non-intrusively, and over prolonged periods of time.

Until very recently, Global Positioning System (GPS) data, despite its ubiquity in mobile smartphone devices and its potential significance to physical location-based avoidance behaviors, has not been leveraged within PTSD-specific research endeavors. Given its wide availability, low resource intensity, and clinical relevance, GPS movement data is a promising novel digital biomarker for the prediction of PTSD diagnostic status and therefore warrants further attention. As such, the current work aimed to utilize the data collected from a pioneering comparative study that investigated the associations between GPS markers of temporal and spatial limitations and PTSD diagnostic status.

For the first time within the PTSD literature, a research group explored patterns of movement among 228 women to discern differences among those with PTSD (*N* = 150), those without PTSD but a history of child abuse (i.e., healthy trauma controls [HTC], *N* = 35), and mentally healthy controls without a history of child abuse (healthy controls [HC], *N* = 43)^53^. Using smartphone-based GPS passive monitoring across seven days, Friedmann et al. (2020) conducted a descriptive statistical study to investigate the relationship between PTSD and functional impairment in terms of movement in space and time around home. From the data collected via the movisensXS app^54^ installed on participants’ smartphone devices, the authors created two location-based features: (1) time (minutes) spent away from home per day and (2) maximum radius around home per day. The authors then employed a comprehensive variety of linear mixed effects modeling to predict each feature, gradually incorporating demographic (employment status, living situation, and hometown population) and clinical (depression and health status) covariates, including a main effect based on weekday/weekend time designations, as well as testing interaction terms based on diagnostic group. Among their findings, they discovered that time spent away from home between the PTSD and HTC groups was statistically significant after controlling for covariates with HTC participants spending less time away from home than those in the PTSD group. At first glance, this result may seem contrary to expectation given the relationship between movement and avoidance behaviors; however, it was hypothesized by Friedmann and colleagues that individuals with PTSD may not perceive spending time outside of home per se as dangerous, but rather that being farther distances from home may more readily induce feelings of insecurity. Moreover, this suggests the importance of simultaneously considering patterns in both time and distance to model PTSD avoidance symptomology, as time on its own may not necessarily provide a theoretically consistent operationalization. Perhaps more intuitively, and in line with the above hypothesis, the maximum radius around home per day was found to be statistically significantly smaller in both PTSD and HTC women compared with the HC group. Interestingly, maximum movement radius for both PTSD and HTC groups was smaller on the weekends compared to their own movement during the weekdays; however, there was no significant difference between these two groups (either in general or within the weekend)^53^.

Taken together, their results implicate GPS movement data as a potentially useful digital biomarker of trauma experience and PTSD within a healthy population of women. However, it is important to note that the results are not reflective of a clear distinction in signal between PTSD individuals and those with a history of child abuse. The work of Friedmann et al. (2020) shows that there is a conflation between the psychopathological correlates of PTSD and the long-term consequences of traumatic experience as concerns movement in daily life. In total, their findings offer a solid empirical argument for GPS-based analytics within the PTSD literature and entice further investigation into the predictive capabilities of GPS information, especially in terms of delineating emotional trauma from PTSD symptomology.

Conducting analyses within a descriptive statistical framework affords an ability to interpret and give meaning to observed trends. Indeed, the Friedmann et al. (2020) study’s use of linear mixed effects modeling was appropriate for an exploratory endeavor that aimed at investigating associations between PTSD and activity space, and the results gave credence to GPS passive sensing data as a detectable, differentiable, and potentially appropriate digital proxy for movement-based behavioral differences in a clinical population with a history of trauma. At this juncture, one new question deals with the legitimacy of further application and whether or not the recently found significance of passive location data may be utilized within a practical, predictive paradigm for diagnostic benefit. Although it sacrifices interpretability for practicality, machine learning approaches have proven to be frequent and successful tools for benchmarking the predictive efficacy of proposed biomarkers within the mental health research space including those derived from passive sensing data^15–17,20–22,26,27,33,55,56^.

One unique strength of these algorithms is their ability to operate within high-dimensional space and consider myriad variables simultaneously. Where linear models, for example, do not scale well with large numbers of predictors, a machine learning model is capable of processing associations and interactions among many (e.g., > 50) variables with relative computational ease. To this point, machine learning algorithms permit useful derivations of novel predictors (i.e., feature engineering) from baseline data to differentially capture the phenomenon in question. In addition, the structure of training, validating, and testing machine learning models ensures that the representative samples used for training are not utilized when predictions are made. This externally validated approach is powerful because it mitigates overly optimistic results and allows for a greater degree of generalizability in performance. Furthermore, if opting to employ an ensemble framework, the pipeline is especially beneficial because it allows for multiple independent and unique learners that parse and evaluate the data differently, thereby capitalizing on the strengths of some algorithms while simultaneously compensating for the weaknesses in others. The final ensemble model therefore bases its predictions on a confluence of all other models whose predictions were differentially informed. Where Friedmann et al. (2020) established the feasibility of applying GPS-based information within the trauma/PTSD research arena with their traditional modeling approach, the current work aimed to leverage the unique affordances of machine learning analytics to further interrogate these location-based digital phenotypes within the predictive domain.

Practically speaking, passive sensing modalities are poised to overcome some of the limitations of traditional self-report and interview-based symptom assessments to ultimately aid clinicians in the diagnostic process. Toward this goal of adding to the diagnostic toolkit, models are built and benchmarked using expert-based metrics (i.e., validated clinical diagnostic outcomes) as the ground truth. To further ensure synergy with clinical practice and maximize potential benefit, careful selection of passive sensing-based markers that serve as theoretically defensible surrogates of the behavior is paramount. In the case of PTSD, location-based digital phenotypes are of notable relevance as they may reflect manifestations of the avoidance symptom cluster and so complement a key aspect of established phenomenology.

**Figure 1:**
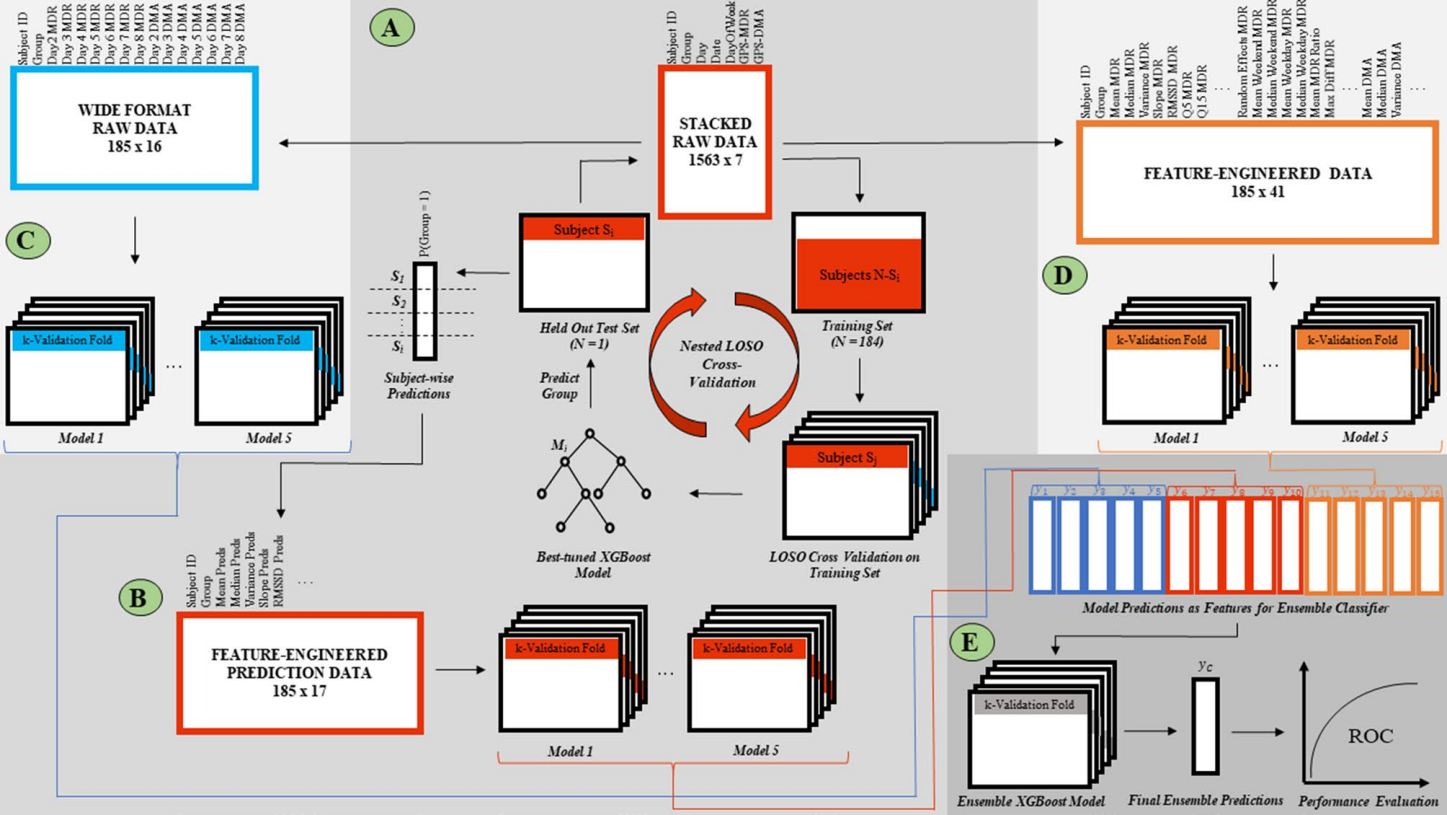
Analytical pipeline. *Note* (**A**) Long format GPS data consisting of two variables – maximum daily radius (MDR) and daily minutes away (DMA) is applied to a nested leave-one-subject-out (LOSO) cross validation machine learning pipeline to train and hyperparameter tune *N* = 185 independent nomothetic xgbDART models in the prediction of subject-wise group membership (1 = child abuse with PTSD; 2 = child abuse with no PTSD). (**B**) Given the stacked format of the raw data, there are multiple subject-wise prediction probabilities equal to the number of days in which GPS data was available for each individual. Thus, distributional features of these prediction probabilities were engineered to form a derived feature space used to train five lower-level machine learning models within a k-fold repeated cross validation sampling methodology. (**C**) The long format GPS raw data is converted to wide format and applied to five machine learning models with k-fold repeated cross validation. (**D**) 39 features are created from the original day-based GPS movement data. The resulting feature space is used to predict group membership with five lower-level machine learning models within a k-fold cross validation framework. (**E**) The resulting prediction probabilities of the fifteen lower-level models in (**A**), (**C**), and (**D**) are used as features in an ensemble xgbDART machine learning model using k-fold repeated cross-validation. The final prediction probabilities of this model are used to statistically evaluate performance in this binary classification task.

Building off of the research conducted by Friedmann et al. (2020) and using data derived from this challenging differential diagnosis (i.e., trauma exposed control subjects vs. those with PTSD), the current study investigated the utility of passively collected, time-anchored location data for the prediction of PTSD diagnostic status. There is increased clinical utility in identifying PTSD within a higher risk population, thus this work focused only on women with a documented history of childhood abuse (PTSD and HTC groups). In particular, this is important as it has the greatest potential utility in aiding in differential diagnosis, and consequently non-trauma exposed controls without a history of abuse (HC) were not considered. From this subcohort, the study developed a novel ensemble modeling pipeline (Fig. 1) to investigate the feasibility of predicting women with PTSD from HTC individuals given only two GPS variables collected passively across seven days. Given the centralized importance of avoidance behaviors in PTSD, the clinical relevance of location data, the combination of the results of the Friedmann et al. (2020) study, and the documented performance of machine learning models across a variety of mental health constructs^57^, this research hypothesized that the constructed modeling pipeline would be capable of discriminating PTSD diagnostic status from traumatized controls with high accuracy (AUC > 0.72), corresponding to a large effect size (Cohen’s d > 0.8)^58^.

## Results

**Figure 2:**
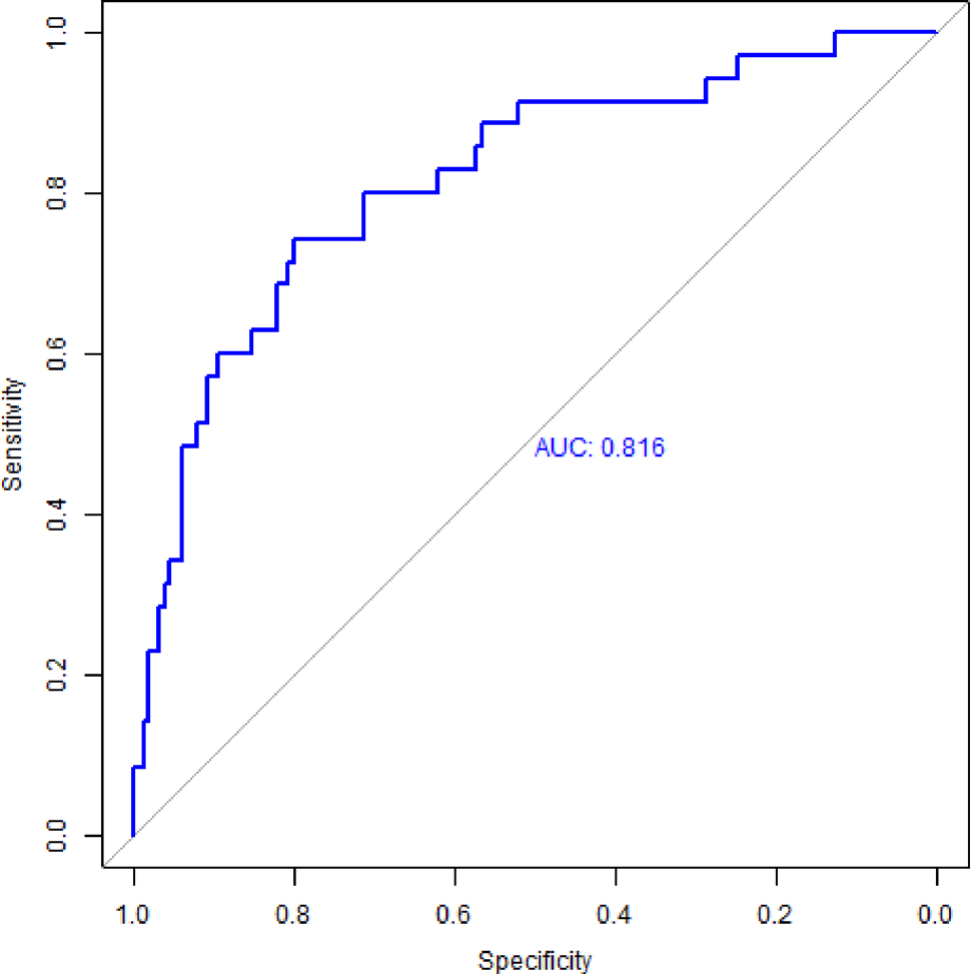
Model Performance ROC. *Note* Ensemble model performance reflects an AUC of 0.816 (accuracy = 0.806, balanced sensitivity = 0.743, balanced specificity of 0.80, Cohen’s kappa = 0.415).

Starting with only two baseline GPS metrics, daily minutes spent away from home (DMA) and maximum daily radius traveled around home (MDR), the proposed ensemble machine learning construct was capable of utilizing a wide assortment of distributional, temporally-relevant, and nomothetic-prediction-based engineered features of passive sensing location information to great effect. Indeed, the binary classification pipeline presented in this work (Fig. 1) reflected a strong ability to discern PTSD diagnostic status in a population of women with a history of child abuse. Across this cohort of *N* = 185 women, the model attained an accuracy of 0.806 (balanced sensitivity = 0.743, balanced specificity = 0.8, balanced accuracy = 0.771) with a corresponding Cohen’s kappa = 0.415 and an AUC = 0.816 (Fig. 2).

## Discussion

The present study aimed to test the efficacy of passively collected, GPS-based location data for the prediction of PTSD diagnostic status in a high-risk cohort with a history of trauma. With only two primary movement variables collected across seven days, the developed ensemble machine learning model was capable of determining clinically validated (Structured Clinical Interview for the DSM-IV [SCID]^59^) PTSD diagnosis with high accuracy. Importantly, this approach offered the first application of GPS passive sensing information within the PTSD prediction literature, thereby providing empirical support for movement data as a behaviorally relevant digital phenotype that characterizes the broader symptomology of PTSD. Moreover, the demonstrated performance, given only a single sensing channel, speaks promisingly to the broader clinical utility of passive sensing information as well as the application of ensemble machine learning frameworks to meaningfully process such data. Where the results of Friedmann et al. (2020) indicated more appreciable differences between PTSD and HTC groups in daily time spent away from home compared to daily maximum distance traveled from home, both of these GPS features were found to be influential to the diagnostic status predictions (PTSD vs. HTC) made within the current work’s machine learning pipeline. For further comparison, it is worth appreciating that the constructed modeling pipeline was capable of correctly classifying PTSD here with greater agreement (kappa = 0.42) than that reflected by the inter-reliability rates of independent raters who evaluated major depressive disorder (kappa = 0.28) or generalized anxiety disorder (kappa = 0.20) in the DSM-5 field trials^60^. This suggests that the current investigation could have great potential for clinical utility.

An additional strength of the analysis borrows from the constituency of the sample population. Prediction of PTSD diagnostic status within a cohort of individuals that had experienced trauma in childhood is a more difficult task compared with prediction among healthy controls with no trauma. Although persons with PTSD experienced a significantly greater magnitude of trauma (as determined via the Childhood Trauma Questionnaire), the score within the trauma exposed control group without PTSD was still appreciable (μ = 59.3 ± 15.9 out of a possible total of 125 points). Moreover, this indicated that even though the PTSD group experienced more severe trauma, the trauma experienced by the non-PTSD group was still substantial. Taken together, the results are particularly striking given that they illustrate a detection of behavioral nuance against a backdrop of past trauma—a clinically relevant scenario where the constellation of requisite PTSD symptoms may be less obvious without more rigorous clinical testing and monitoring.

Indeed, results showed that without reliance on any clinical predictors, passive monitoring alone could correctly diagnose PTSD approximately 81% of the time. Where digital biomarker research for PTSD has already shown strong support for HRV, skin conductance, and linguistic features as meaningful signals for this disorder within diagnostics and screening, the present findings encourage integration with GPS data toward testing of a holistic digital fingerprint for PTSD predictive tasks. Importantly, the passive sensing approach offers benefits that can complement and enhance in-person assessments. First, the GPS passive sensing stream can be viewed as a potential add-on to current clinically validated measures that may aid practitioners in flagging PTSD symptom states and/or deterioration for subsequent expert treatment. Since PTSD symptom manifestations and magnitudes are known to fluctuate over time, this methodology affords an opportunity to capture longitudinal, in-the-moment changes that can be more acutely actionable by medical professionals. Second, such a passive monitoring paradigm would not require additional active burden or time commitment from patients. In consideration of the wait times that patients typically experience when seeking and scheduling initial and follow-up appointments, quick installation of a smartphone app that can meaningfully track and parse a patient’s movement profile in the meanwhile makes use of treatment delays while potentially assisting the healthcare professional once the patient is able to be seen. Overall, in practical application, the temporally sensitive detection of physio-behavioral deterioration within a mobile, minimally invasive paradigm could be integrated with just-in-time adaptive digital interventions to ultimately promote clinical help-seeking and in-person psychiatric treatment. After further refinement and testing in diverse cohorts, deployment of this modeling framework could be realized as an easy-to-use smartphone app that collects, summarizes, and sends information to healthcare providers, thereby programmatically generating PTSD movement-based profiles for their patients.

This work has a few important limitations. The entirely female representation in the study population, while important from the context of the limited literature that currently exists at the crossroads of PTSD and passive sensing, nonetheless restricts the generalizability of the results. It is unclear whether or not the behavioral patterns detected by the machine learning algorithms are representative of men or those of either sex suffering from disparate traumas. In a similar vein, the distal nature of the trauma and the varying amount of time that has elapsed since the trauma, represent potential confounders. Additionally, diagnostic predictions were based on a short window of assessment (one week), thus the stability of the model’s performance within more robustly longitudinal contexts is unknown.

One theoretical and practical challenge of studying and diagnosing PTSD stems from its high comorbidity rates with other psychiatric disorders (e.g., major depressive disorder). From a predictive modeling standpoint, this study was able to demonstrate appreciable concordance with clinically determined PTSD status among women with a prior history of child abuse; however, the current work was unable to determine whether the informative patterns of movement detected by the model were a reflection of behavioral characteristics that stem primarily from PTSD itself or from related comorbidities such as an anxiety or mood disorder. An ability to control for these comorbidities in future modeling efforts will strengthen generalizability and more rigorously test diagnostic specificity towards potential clinical application. Larger cohorts with a more comprehensive collection of related mental health metrics will allow for more precise evaluation.

Of final note, the advantages of machine learning models over traditional statistical approaches are well-documented empirically (and mentioned above), yet interpretability has traditionally been its Achilles heel. Without use of fairly recent introspection methods^61^, the ability to understand how the model makes decisions and the relative contributions of predictors is limited. The current results of this study, however preliminary, pave the way for future replication and refinement across larger and disparate sample populations. The advent of smart devices, advanced sensor technologies, and complex learning algorithms promotes research into the discovery, testing, and application of new digital correlates for old behaviors. Within the mental health space, this equates to the development of risk factors that are no longer bounded by the practical and theoretical measurement limitations of a clinical environment, aiding both patient and clinician alike in their struggle with illness.

## Methods

### Study population and original dataset

This work utilized data collected from a recent study that was interested in further investigating the association between childhood (before the age of 18) sexual or physical abuse and functional impairment in later adult life^53^. In this research, the authors leveraged smartphone-based GPS passive sensing technology to capture the temporal and spatial movement patterns of *N* = 228 German women over the course of seven days. Using GPS data as a digital surrogate for functional impairment, women diagnosed with PTSD by the Structured Clinical Interview for the DSM-IV (SCID)^59^ subsequent to a documented history of child abuse (*N* = 150, μ_age_ = 35.5 years, employment rate = 49.3%, living alone = 30%, μ_GAF_ = 49.6) were compared to those assessed as mentally healthy with (*N* = 35, μ_age_ = 32.1 years, employment rate = 71.4%, living alone = 48.6%, μ_GAF_ = 88.9) and without (*N* = 43, μ_age_ = 32.3 years, employment rate = 55.8%, living alone = 19%, μ_GAF_ = 91.4) past experiences of child abuse. Age, employment, and living situation were not found to be statistically significant between groups; however, statistically significant (*p* < 0.001) differences existed for all clinical characteristics of both psychosocial functioning (including the Global Assessment of Functioning Scale [GAF])^62^ and PTSD severity. Additional information regarding recruitment procedures, clinical indices, and demographic variables are provided in the original article^53^.

All methods concerning the acquisition of data utilized in the current analysis were carried out in accordance with relevant guidelines and regulations. Written informed consent for participation was obtained, and all associated protocols were approved by the following three ethics committees: (1) Ethik-Kommission II of the Medical Faculty of the Central Institute of Mental Health, Mannheim and Heidelberg University, (2) Ethikkommission of the Psychologisches Institut of the Humboldt-Universitat zu Berlin, and (3) Ethikkommission of the Fachbereich für Psychologie und Sportwissenschaften of the Goethe University Frankfurt (study reference number: 2013-635 N-MA).

### Outcome metric

As the current research was particularly interested in the potential ability to discriminate PTSD status within a higher risk population of prior abuse (a more difficult task than the identification of PTSD across healthy controls without a previous history of abuse), only GPS data corresponding to women diagnosed with PTSD and a history of child abuse (Group = 1) and women determined to have had a prior history of abuse without a concomitant diagnosis of PTSD (Group = 2) were analyzed in this work. Accordingly, the outcome metric of interest concerns binary designation of group status for a subcohort of *N* = 185 women.

### Baseline GPS variables

The GPS data provided by Friedmann et al. (2020) consists of two continuous variables collected over a seven-day period. Daily minutes spent away from home (DMA) and the maximum daily radius around home (MDR) were calculated using distances derived from the spatial difference between subsequent measures of latitude and longitude. Latitude and longitude coordinate differences were computed separately with subsequent application of the Pythagorean Theorem to determine the final distance vector (in kilometers). Natural inconsistencies in distance across longitudes were corrected. The designation of “home” was based on a radius of 500 m around the geo code specified by participants as their home location. The day-anchored DMA and MDR comprise the stacked raw GPS data utilized in the current study (Fig. 1A).

### Machine learning model types

The machine learning pipeline constructed for this work utilized several distinct model types available via the R *caret* package^63^ to capitalize on the unique strengths available through differences in algorithmic approaches and decision criteria. For each “arm” of the analytical pipeline (Fig. 1B–D), five lower-level classifiers were used: (1) an extreme gradient boosting machine with deep neural net dropout techniques (xgbDART)^64,65^, (2) a partial least squares regression model^66^, (3) a lasso-regularized generalized linear model^67^, (4) a support vector machine with a radial basis kernel^68^, and (5) a conditional inference random forest algorithm^69^. Explicit use of these models will be noted in subsequent sections.

### Data preprocessing

All baseline predictors and engineered features were individually standardized prior to model application such that data had μ = 0 and σ = 1. Missing data generated as a consequence of wide-formatted temporal data was imputed by the mean across subjects within the respective time-based feature. This concerned five models from the analytical pipeline (Fig. 1C).

### Machine learning pipeline—Arm 1

#### Stacked baseline GPS data and nested LOSO modeling

The first part of the ensemble machine learning analysis (Fig. 1A) only concerned the original DMA and MDR variables. For this phase of the pipeline, *N* = 185 independent, nomothetic xgbDART models were trained, hyperparameter tuned using grid search, and validated within a nested leave-one-subject-out (LOSO) cross validation framework to predict group membership on the held-out participant. Note that because of unequal class representation in the data (~ 80% Group 1, ~ 20% Group 2), the popular Synthetic Minority Oversampling Technique (SMOTE)^70^ was implemented as a built-in functionality in *caret*. Briefly, SMOTE is an effective and robust method of creating novel synthetic examples from the minority class within the training set that are plausible and relatively close in feature space to existing examples within the minority class. The resulting output of each of these models consisted of prediction probabilities associated with subject-wise membership in Group 1. Because the raw GPS data was organized such that multiple measures were available for each subject, i.e., each row of data was equivalent to a DMA and MDR value that represented that day (for a total of 3–7 rows/days a subject participated in the study), there were multiple prediction probabilities (3–7) computed by the models for each subject. Taken together, this step created a univariable dataset of Group 1 prediction probabilities for all *N* = 185 subjects based on a nomothetic, out-of-fold modeling paradigm.

#### Feature engineering of the nested LOSO prediction space

Using the nested LOSO-based model predictions from the previous step (Fig. 1A), the study then calculated descriptive, distributional statistical features for use downstream (Fig. 1B). Here, 15 new variables were defined for each subject based on the (i) mean, (ii) median, (iii) variance, (iv) slope, (v) root mean square of successive differences (RMSSD), and (vi–xv) quantiles ranging from 5 to 95% in 10% increments of their associated nested LOSO model predictions.

#### Modeling of the derived LOSO prediction-based features

Derived predictors from feature engineering of the nested LOSO predictions were then applied to the five aforementioned machine learning models, each in a ten-fold, five-times-repeated, cross-validation framework with grid-based hyperparameter tuning and SMOTE, to predict group membership. The resulting Group 1 model prediction probabilities for each of these five models were saved for use in the final ensemble model (Fig. 1E, red).

### Machine learning pipeline—Arm 2

#### Wide format baseline GPS data

The stacked raw GPS data utilized at the beginning of Arm 1 was converted to wide format which resulted in fourteen day-based variables, seven corresponding to DMA and seven corresponding to MDR, representing the maximum seven-day length of data collection (Fig. 1C).

#### Modeling of the time-anchored baseline GPS data

With the wide-formatted DMA and MDR data, the five machine learning models mentioned above were trained and validated using ten-fold, five-times-repeated cross validation with grid search hyperparameter tuning and SMOTE, to predict group membership (Fig. 1C). The resulting Group 1 model prediction probabilities for each of these five models were saved for use in the final ensemble model (Fig. 1E, blue).

### Machine learning pipeline—Arm 3

#### Feature engineering of the stacked baseline GPS data

From the baseline GPS data in stacked format, 39 new variables were derived that capitalized on the distributional statistics, subject-specific random effects, and time-anchored weekday/weekend context of both MDR and DMA. For MDR these included: (i) overall mean, (ii) overall median, (iii) overall variance, (iv) slope, (v) root mean square of successive differences (RMSSD), (vi–xv) quantiles ranging from 5 to 95% in 10% increments, (xvi) subject-specific random effects from an intercept-only linear mixed effects model using the *lme4* package^71^, (xvii) weekday mean, (xviii) weekend mean, (xix) ratio of mean weekday to mean weekend, and (xx) difference in the maximum weekend MDR from the maximum weekday MDR. For DMA, the first 18 listed above for MDR were reiterated (xxi–xxxviii). In addition, (xxxix) the percent of time spent away from home on the weekend as a function of the total time spent away from home across all days was calculated.

#### Modeling of the feature-engineered stacked baseline GPS data

Through application of the derived 39-feature space of the stacked baseline GPS data, the five machine learning models mentioned previously were trained and validated using ten-fold, five-times-repeated cross validation with grid search hyperparameter tuning and SMOTE, to predict group membership (Fig. 1D). The resulting Group 1 model prediction probabilities for each of these five models were saved for use in the final ensemble model (Fig. 1E, orange).

#### Machine learning pipeline—ensemble

Group 1 prediction probabilities from the fifteen lower-level models illustrated in Arm 1 (Fig. 1B), Arm 2 (Fig. 1C), and Arm 3 (Fig. 1D) were used as predictors in a final xgbDART machine learning model (Fig. 1D). The xgbDART model (an extension of the xgBoost algorithm) was selected as the final machine learning model because of its often-cited high performance and execution speed in a variety of machine learning tasks. Briefly, xgBoost operates by constructing decision trees in a sequential manner where each subsequent tree in the sequence learns from the mistakes of its predecessor and updates the residual errors accordingly. This process of boosting converts what would at baseline be a set of weak learners into one final strong learner. The choice to use xgbDART over the baseline xgBoost provided the additional feature of random dropout where boosted trees are randomly removed throughout the learning process. Drawing from practice in neural network modeling, this mitigates the possibility of overfitting or “overspecialization” caused by the first few trees in the boosted sequence dominating model performance^64^. As in all of the lower-level models, prediction of group membership was carried out within a ten-fold, five-times-repeated cross validation framework with grid search hyperparameter tuning and SMOTE. The prediction probabilities from this ensemble model represent the final output of this binary classification machine learning analytical pipeline.

### Model evaluation

The final ensemble machine learning model was primarily assessed for performance based on parameters of the Receiver Operating Characteristic (ROC) curve. Sensitivity (true positive rate), specificity (true negative rate), and the Area Under the Curve (AUC) are key components of the ROC that quantify model tradeoffs in the true positive and true negative rates over a continuum of decision thresholds or cut points. In particular, the balanced sensitivity and specificity are reported as these correspond to the threshold that represents the optimal tradeoff. This study also reports raw model accuracy, balanced accuracy (the average of the sum of the sensitivity and specificity), and Cohen’s kappa. The *caret* and *pROC*^72^ packages in R (v4.0.2) were used to calculate and visualize these results. Additionally, performance results for each of the 15 lower-level models of the ensemble pipeline are provided for reference in Supplementary Table 1.

## Supplementary Information

Supplementary Information 1.
