# Supplementary material for: Using artificial intelligence and longitudinal location data to differentiate persons who develop posttraumatic stress disorder following childhood trauma
Source: Sci Rep. 2021 May 13;11:10303. doi: 10.1038/s41598-021-89768-2 (PMC8119967; doi:10.1038/s41598-021-89768-2)
Supplement: Supplementary file 1 — Supplementary Information 1. [file 41598_2021_89768_MOESM1_ESM.docx]

Supplemental Table 1. Baseline Model Performance Summary

| **Model** | **Pipeline Arm** | **Accuracy** | **Sensitivity** | **Specificity** | **Cohen’s Kappa** | **AUC** |
| --- | --- | --- | --- | --- | --- | --- |
| xgbDART | 1 | 0.723 | 0.743 | 0.720 | 0.247 | 0.70 |
| pls | 1 | 0.704 | 0.800 | 0.547 | 0.153 | 0.64 |
| glmnet | 1 | 0.703 | 0.686 | 0.533 | 0.137 | 0.61 |
| cforest | 1 | 0.726 | 0.743 | 0.640 | 0.235 | 0.68 |
| svm | 1 | 0.800 | 0.543 | 0.673 | -0.010 | 0.58 |
| xgbDART | 2 | 0.707 | 0.371 | 0.840 | 0.153 | 0.62 |
| pls | 2 | 0.670 | 0.314 | 0.793 | 0.060 | 0.54 |
| glmnet | 2 | 0.642 | 0.771 | 0.407 | 0.073 | 0.58 |
| cforest | 2 | 0.758 | 0.114 | 0.947 | 0.052 | 0.47 |
| svm | 2 | 0.811 | 0.371 | 0.847 | 0.005 | 0.59 |
| xgbDART | 3 | 0.722 | 0.171 | 0.973 | 0.040 | 0.48 |
| pls | 3 | 0.732 | 0.629 | 0.513 | -0.022 | 0.52 |
| glmnet | 3 | 0.646 | 0.143 | 0.773 | -0.017 | 0.46 |
| cforest | 3 | 0.783 | 0.200 | 0.887 | 0.120 | 0.45 |
| svm | 3 | 0.810 | 0.229 | 0.680 | -0.002 | 0.43 |

*Note.* xgbDART = extreme gradient boosting machine with deep neural net dropout techniques; pls = partial least squares regression; glmnet = lasso-regularized generalized linear model; cforest = conditional inference random forest; svm = support vector machine with radial basis kernel; See Methods section and Figure 1 for details on the three Arms of the analysis pipeline.
